# A Remarkable Response of Granulomatous Hypophysitis to Infliximab in a Patient With a Background of Crohn's Disease—A Case Report

**DOI:** 10.3389/fendo.2020.00350

**Published:** 2020-05-29

**Authors:** Bahar K. Force, Tiphanie P. Vogel, Dang M. Nguyen, Kent A. Heck, Sherly Sebastian, Mas Takashima, Daniel Yoshor, Susan L. Samson

**Affiliations:** ^1^Section of Endocrinology, Diabetes and Metabolism, Department of Medicine, Baylor College of Medicine, Houston, TX, United States; ^2^Baylor St. Luke's Pituitary Center, Houston, TX, United States; ^3^Division of Rheumatology, Department of Pediatrics, Baylor College of Medicine, Houston, TX, United States; ^4^Greater Houston Gastroenterology, Houston, TX, United States; ^5^Department of Pathology and Immunology, Baylor College of Medicine, Houston, TX, United States; ^6^Department of Neurosurgery, Baylor College of Medicine, Houston, TX, United States; ^7^Houston Methodist Hospital, Department of Otolaryngology, Houston, TX, United States

**Keywords:** pituitary, granulomatous hypophysitis, inflammatory bowel disease, Crohn's disease, anti-TNF-alpha, infliximab, adalimumab, case report

## Abstract

**Background:** Hypophysitis is primary or idiopathic or secondary to another disease process. The histologic subtypes of hypophysitis are lymphocytic, granulomatous, xanthomatous, xanthogranulomatous, or IgG4-related. Granulomatous hypophysitis is the second most common form and is characterized by multinucleated giant cells with granulomas and histiocytes. It can be idiopathic or secondary to another process such as infection, sarcoidosis, vasculitis, dendritic cell disorders, Crohn's disease (CD) or a reaction to rupture of a Rathke's cyst or pituitary adenoma. We present a case of granulomatous hypophysitis in a patient with CD who had resistance to corticosteroids but a dramatic response to immunosuppressive therapy with anti-tumor necrosis factor (TNF)-α therapy.

**Case description:** A 43-year-old woman with a 9-year history of ileal and colonic CD presented to the Pituitary Center with headaches, visual disturbance, fatigue, nausea, and secondary amenorrhea. She was not on active therapy for her CD at the time of presentation and had no gastrointestinal symptoms. Hormonal evaluation revealed hyperprolactinemia, secondary hypothyroidism and adrenal insufficiency. MRI revealed a 12 × 12 × 19 mm sellar lesion abutting the optic chiasm, reported as a macroadenoma. The patient underwent endoscopic transsphenoidal biopsy of the pituitary mass. Pathology revealed granulomatous hypophysitis. Evaluation for secondary causes of hypophysitis, apart from CD, was negative. Despite a course of high dose prednisone, her symptoms and MRI findings worsened and she developed symptoms consistent with diabetes insipidus. Using a personalized medicine approach, she was started on anti-(TNF)-α therapy with infliximab combined with azathioprine, which are indicated for treatment of CD. Her headaches and polyuria resolved and her menstrual cycles resumed. MRI at 3 months and more than 1.5 years after initiation of anti-TNF-α therapy revealed durable resolution of the pituitary mass.

**Conclusion:** To our knowledge, this is the first report of successful use of anti-TNF-α therapy for a patient with granulomatous hypophysitis, in this case associated with a previous diagnosis of CD. Although glucocorticoids are used frequently as first-line therapy for primary hypophysitis, granulomatous hypophysitis can be corticosteroid resistant and other immunosuppressive approaches may need to be considered within the context of the patient.

## Introduction

Hypophysitis is defined as an acute or chronic inflammation of the pituitary gland. It is an uncommon pituitary disorder with estimated annual incidence of 1 in 9 million surgical cases (1). The inflammation can be localized to the anterior pituitary (adenohypophysitis), the pituitary stalk and posterior pituitary (infudibuloneurohypophysitis), or it can involve the pituitary gland in its entirety (panhypophysitis) (2). The clinical manifestations can include anterior pituitary hormone deficiencies, hyperprolactinemia, and diabetes insipidus (DI) (3). Patients frequently present with headaches, but they may also have visual disturbances due to mass effect of the inflamed and enlarged pituitary gland on the optic chiasm or cranial nerves II, III, IV, and/or VI (2, 3). Dedicated gadolinium-enhanced pituitary magnetic resonance imaging (MRI) can reveal a symmetrically enlarged and homogenously enhancing pituitary gland, infundibular thickening, and loss of the posterior pituitary bright spot (4).

Histologic inspection allows for classification of hypophysitis as lymphocytic (predominantly lymphocytes), granulomatous (multinucleated giant cells with granulomas and histiocytes), xanthomatous (lipid filled “foamy” macrophages with granulomas), xanthogranulomatous (mixed histology), plasmacytic (IgG4 positive plasma cells) and, very rarely, necrotizing hypophysitis (2). Primary hypophysitis denotes an autoimmune, inflammatory involvement of the gland as an isolated or idiopathic finding while secondary hypophysitis is a sellar manifestation of a systemic disease or reaction to a local lesion (2). Granulomatous hypophysitis (GrHy) is the second most prevalent form of hypophysitis, after lymphocytic, and the disease processes reported to be associated with secondary GrHy include infection (tuberculosis, syphilis, mycosis), sarcoidosis, granulomatosis with polyangiitis, Takayasu arteritis, Cogan's syndrome, dendritic cell disorders (Langerhans Cell Histiocytosis, Erdheim Chester), or a local sellar lesion (Rathke's cyst, pituitary adenoma, germinoma, Craniophayringioma) (5, 6). Here we present a patient case of GrHy associated with Crohn's disease (CD), with resistance to corticosteroids but a dramatic response to anti-tumor necrosis factor (TNF)-α antibody therapy combined with azathioprine.

## Case Report

A 43-year-old woman with a past medical history of ileal and colonic CD for 9 years was referred to the Pituitary Center for a sellar mass. Written informed consent was obtained from the patient to report the details of her case. She had been experiencing intermittent debilitating headaches associated with photophobia, diplopia and subjective loss of peripheral vision for 9 months. During one of these episodes, she presented to an outside emergency room where MRI of the brain reportedly showed a pituitary macroadenoma. She was also seen by an ophthalmologist, and underwent formal visual field testing which was normal. Review of systems was positive for worsening fatigue, nausea and secondary amenorrhea for 5 months. She denied symptoms of polyuria or polydipsia. Her CD was considered to be in remission off of medications, but she had been on mesalamine 1 year prior, and corticosteroids were last prescribed 2 years ago. She had no family history of endocrinopathies or pituitary tumors. Her physical examination was notable for a sallow complexion, but was otherwise unremarkable.

**Figure 1:**
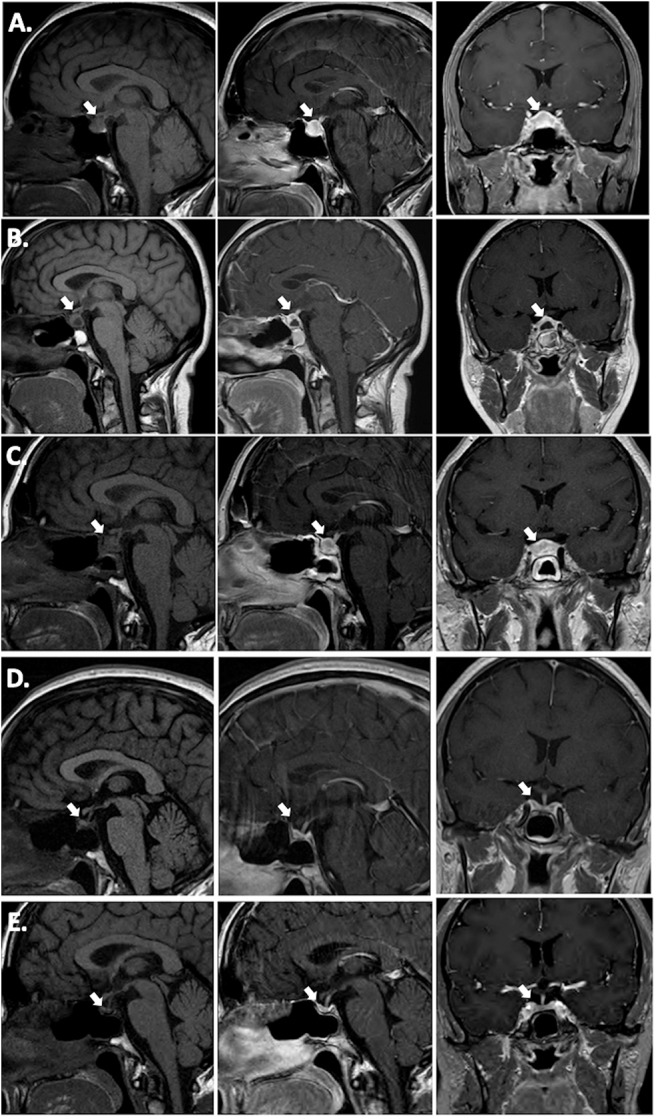
T1 pre-contrast sagittal images (left column), T1 post-contrast sagittal images (center column) and T1 post-contrast coronal images (right column). **(A)** Pre-operative; **(B)** 2 weeks post-operative; **(C)** 15 weeks (3.5 months) post-operative, 3 weeks post-corticosteroid taper; **(D)** 26 weeks (6 months) post-operative, 12 weeks post-TNF-α inhibitor initiation (infliximab) immediately prior to azathioprine addition; **(E)** 17 months post-TNF-α inhibitor (infliximab combined with azathioprine for 15 months, followed by adalimumab combined with azathioprine).

Hormonal testing revealed secondary hypothyroidism with a low TSH of 0.271 (0.45–4.5 μIU/mL) and low free T4 of 0.57 (0.82–1.77 ng/dL), secondary adrenal insufficiency with a low cortisol of 1.5 μg/mL with an inappropriately normal ACTH of 8 (6–58 pg/mL), and mildly elevated prolactin of 62.2 (4.8–23.3 ng/mL). There was no symptomatic or laboratory evidence of diabetes insipidus (DI) and her urine osmolality was 651 mOsm/kg concurrent with a serum sodium of 141 mEq/L. MRI brain revealed a 12 mm anterior-posterior x 12 mm craniocaudal x 19 mm transverse sellar lesion abutting the optic chiasm. It was reported as a pituitary adenoma, but infundibular thickening was visible upon closer examination (Figure 1A). The presumed posterior pituitary “bright spot” was visible on the MRI T1 pre-contrast sagittal view (Figure 1A, *left panel*).

**Figure 2:**
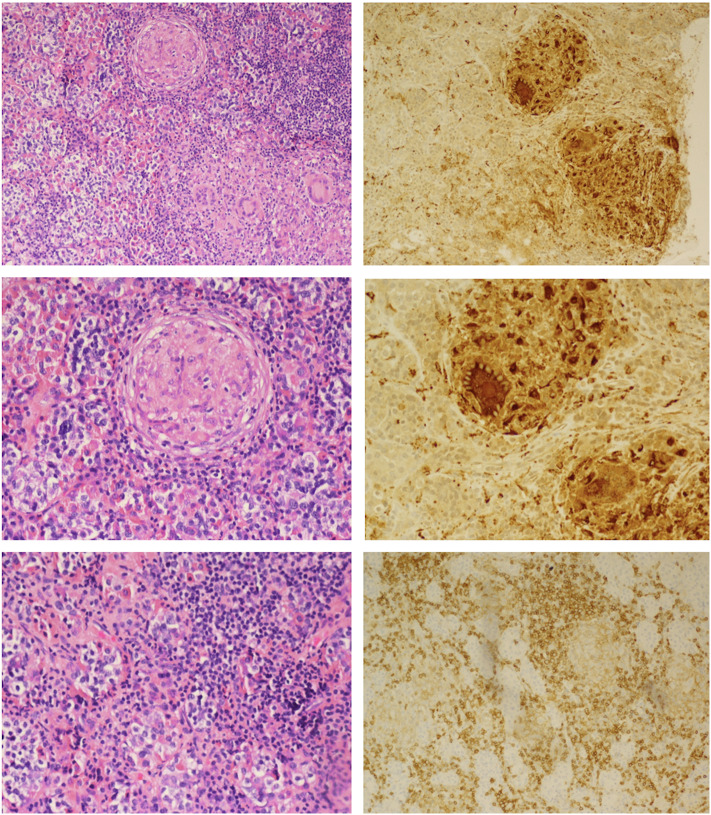
Hematoxylin and eosin staining (Left): Upper, non-caseating granulomatous inflammation interrupting adenohypophyseal glandular cytoarchitecture; Middle, higher magnification showing multinucleate giant cells; Lower, lymphohistiocytic inflammation and fibrosis. Immunoperoxidase staining (Right): Upper and Middle, anti-CD68 macrophage marker highlighting macrophage and multinucleated giant cells; Lower, anti-CD45 lymphoid marker for lymphocytes.

The patient was initiated on levothyroxine and hydrocortisone replacement. She underwent endoscopic transsphenoidal biopsy and partial resection of the pituitary lesion, with the surgical cavity visible on the post-operative MRI (Figure 1B). The first five surgical specimens contained adenohypophysis with lymphocytic infiltrates (anti-CD45 positive) and multiple foci of epithelioid histiocytes with focal multinucleation (anti-CD68 positive) as shown in Figure 2. The sixth specimen was a dural biopsy showing fibroconnective tissue consistent with dura, with mild chronic inflammation.

Pathology was consistent with a diagnosis of GrHy (Figure 2). Special stains for fungi, acid-fast organisms and bacterial organisms were negative. Further evaluation for secondary causes of GrHy, apart from CD, including tuberculosis (TB), syphilis, vasculitis and sarcoidosis was negative (Table 1). Additionally, there was no clinical or histological suspicion for dendritic cell disorders such as Langerhans cell histiocytosis or Erdheim-Chester disease.

**Table 1:** Laboratory evaluation for secondary causes of granulomatous hypophysitis.

**Laboratory test**	**Reference range**	**Patient result**
Anti-nuclear antibody screen	Negative	Positive
Erythrocyte sedimentation rate	0–20 mm/hour	35
Quantiferon TB Gold	Negative	Negative
Syphilis RPR screen	Non-reactive	Non-reactive
Anti-neutrophil cytoplasmic autoantibodies	<1:10	<1:10
Perinuclear anti-neutrophil antibodies (Myeloperoxidase antibody)	≤20 unit	3
Cytoplasmic anti-neutrophil antibodies (Serine protease 3)	≤20 unit	3
Angiotensin converting enzyme	11–80 U/L	33
Thyroid peroxidase Ab	<9 IU/mL	2
IGG-4 subclass	4–86 mg/dL	11

**Figure 3:**
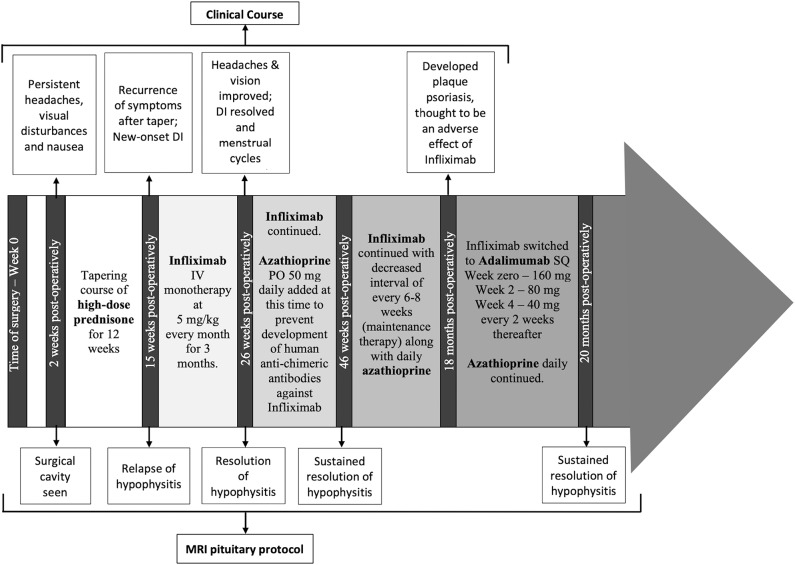
The timeline of the patient's clinical course, treatment and MRI monitoring are shown from the time of surgery (0 weeks) through to the most recent imaging at 20 months from surgery.

A timeline of therapeutic interventions after surgery is depicted in Figure 3. The patient was prescribed high dose oral prednisone, starting at 60 mg daily for 2 weeks, and tapering over 8 more weeks to 5 mg. However, she continued to have a worsening clinical course, including the development of new-onset disruptive polyuria and polydipsia with a serum sodium 142 mEq/L concurrent with a urine Osmolality 320 mOsm/kg, treated successfully with demopressin 0.1 mg daily. MRI revealed relapse of her mass lesion on MRI (Figure 1C).

In conjunction with her specialists in Rheumatology and Gastroenterology, a personalized medicine approach was proposed using an anti-tumor necrosis factor (TNF)-α inhibitor because of the efficacy of these agents in CD. Initially, she was started on infliximab. Within 3 months of therapy, she experienced a marked improvement in symptoms and resolution of the mass on MRI (Figure 1D). Azathioprine was added 3 months after infliximab, as per advice from Rheumatology, to reduce the risk for development of neutralizing human anti-chimeric antibodies against the infliximab, as they decrease its efficacy.

While she has persistent central hypothyroidism and adrenal insufficiency, the symptoms of DI resolved within 4 months of initiation of immunosuppressive therapy, and she does not require desmopressin. She had temporary resumption of regular menstrual cycles as well. However, 6 months later her cycles were again interrupted due to recurrence of mild hyperprolactinemia to 33.2 ng/mL, which was thought to be due to infiltration of the stalk. She was started on low-dose cabergoline 0.25 mg weekly, with resolution of amenorrhea and hyperprolactinemia. Her course has been complicated by development of plaque psoriasis which could be due to her predisposition to autoimmunity, but can also be seen paradoxically as a side effect of infliximab. She was switched to a fully humanized anti-TNF-α inhibitor adalimumab at 18 months post-operatively, and azathioprine was continued (Figure 3). Subsequent MRI at nearly one and a half years after initiation of immunosuppressive therapy, including 2 months on adalimumab, revealed sustained resolution of the pituitary lesion (Figure 1E).

## Discussion

Granulomatous hypophysitis is a rare pituitary disorder that usually presents in the fifth decade of life and has a 3:1 female preponderance (4). Etiologically, it may be primary (idiopathic) or secondary to another process (5, 6). In the current case, testing for multiple secondary causes was negative (Table 1), but the prior diagnosis of CD was intriguing. The histopathologic findings in the bowel for CD include granulomatous lesions, and GrHy has been reported to be associated with CD in three previous cases (7–9).

Compared to lymphocytic hypophysitis, GrHy may be more clinically severe with higher frequency of headaches, visual disturbances and endocrine dysfunction (3). DI may be seen in about 75% of patients (3). Some investigators also propose that GrHy may be a later manifestation of pituitary inflammation following earlier lymphocytic infiltration (6). Therapeutic options for severely symptomatic patients with GrHy include transsphenoidal debulking and glucocorticoids, alone or in conjunction with each other (10). However, as seen in our case, GrHy can be corticosteroid resistant, necessitating a different strategy for immune modulation. Infliximab and rituximab have been used in primary lymphocytic hypophysitis resistant to corticosteroids, while rituximab has been used in one case of primary GrHy (11, 12). Rituximab is a monoclonal antibody against the CD20 receptor on the surface of B-cells and leads to immune-mediated B-cell clearance, thereby affecting the production of antibodies and reducing antigenic presentation to T-cells (12). There are no reports in the literature, to our knowledge, regarding use of anti-TNF-α therapy for GrHy. TNF-α is a proinflammatory cytokine that is a principal mediator of inflammation in CD (13). It is responsible for recruitment and activation of T-lymphocytes at local sites of inflammation. Infliximab and Adalimumab are chimeric (mouse-human) and fully humanized monoclonal antibodies, respectively, which neutralize TNF-α, halting the inflammatory cascade. Additionally, these inhibitors accelerate apoptosis of activated T-lymphocytes (13). These effects induce remission in patients with CD, both in the bowel and for extraintestinal disease, including musculoskeletal and mucocutaneous manifestations (14).

Given the known diagnosis of Crohn's disease in our patient, our rationale for using anti-TNF-α therapy was secondary to the possibility that the mechanism of inflammation involved with CD could also be contributing to the GrHy. This case illustrates that GrHy in association with CD may respond to anti-TNF-α therapy, including with partial restoration of pituitary function. The rapidity of response, within 3 months, and sustainability of remission, even at one and a half years after initiation of anti-TNF-α therapy is striking. Although we cannot extrapolate this case example to primary GrHy, in the absence of CD, it is tempting to speculate that anti-TNF-α agents could be successful in other cases of primary GrHy, given that TNF-α is a key cytokine in granuloma formation and there is broadening use of this therapy in other granulomatous diseases beyond inflammatory bowel disease (15–17). In this era of personalized medicine, this case also highlights the importance of multidisciplinary treatment strategies tailored to the patient to provide optimal care.

## Data Availability Statement

The datasets generated for this study are available on request to the corresponding author.

## Ethics Statement

We have approval by the Baylor College of Medicine Institutional Review for a database of surgical outcomes of pituitary patients. The patients/participants provided their written informed consent to participate in this study.

## Author Contributions

All authors were involved in clinical decision making and care of this patient. BF wrote the initial draft of the manuscript. Editorial changes were made by SLS and TV. KH was the pituitary pathologist and performed the immunostaining and contributed the histology figures.

## Conflict of Interest

The authors declare that the research was conducted in the absence of any commercial or financial relationships that could be construed as a potential conflict of interest.
